# High Acceptability of HIV Self-Testing among Technical Vocational Education and Training College Students in Gauteng and North West Province: What Are the Implications for the Scale Up in South Africa?

**DOI:** 10.1371/journal.pone.0169765

**Published:** 2017-01-31

**Authors:** Mathildah Mpata Mokgatle, Sphiwe Madiba

**Affiliations:** 1 School of Public Health, Department of Biostatistics, Sefako Makgatho Health Sciences University, South Africa; 2 School of Public Health, Department of Environmental and Occupational Health, Sefako Makgatho Health Sciences University, South Africa; The First affiliated Hospital of China Medical University, CHINA

## Abstract

**Background:**

Although HIV self-testing (HIVST) is globally accepted as an important complement to existing HIV testing approaches, South Africa has lagged behind in its adoption. As a result, data on the acceptability and uptake of HIVST testing is limited. The study investigated the acceptability of HIVST among students in Technical Vocational Education and Training (TVET) colleges in two provinces in South Africa.

**Methods:**

A cross-sectional survey using a self-administered structured questionnaire was used to collect data among 3,662 students recruited from 13 TVET colleges.

**Results:**

The mean age of the students was 21.9 years. The majority (80.9%) were sexually active; while 66.1% reported that they had one sexual partner, and 33.9% had two or more sexual partners in the past year, and66.5% used condoms during the last sexual act. Three-quarters tested for HIV in the past year but less than half knew about HIVST prior to the survey. The acceptability of HIVST was high; about three-quarters showed a willingness to purchase a self-test kit and a majority would self-test with partners. Acceptability of HIVST was associated with being sexually active (OR = 1.73, *p* = 0.02, confidence interval (CI): 1.08–2.75), having ever been tested for HIV (OR = 1.74, *p* = 0.001, CI: 1.26–2.38), and having multiple sexual partners (OR = 0.61, *p* = 0.01, CI: 0.42–0.88). Three-quarters would confirm test results at a local health facility. In terms of counselling, telephone hotlines were acceptable to only 39.9%, and less than half felt that test-kit leaflets would provide sufficient information to self-test.

**Interpretations:**

The high acceptability of HIVST among the students calls for extensive planning and preparation for the scaling up of HIVST in South Africa. In addition, campaigns similar to those conducted to promote HIV counselling and testing (HCT) should be considered to educate communities about HIVST.

## Background

The South African National Strategic Plan on HIV, STIs and TB aims to get 80% of the population to know their HIV status by 2016 [1]. However, available HIV counselling and testing (HCT) strategies like client or provider-initiated counselling and testing will not be sufficient to achieve universal access to HIV testing and treatment [2] because of challenges that limit their effect and scope [3, 4]. Additionally, HIV self-testing (HIVST) is globally accepted as an important complement to existing HCT approaches, and several countries including South Africa have already introduced or are considering the introduction of HIVST as part of their testing strategies [2, 5, 6]. Moreover, HIVST has the potential to facilitate more people getting tested because it has advantages of convenience, speed, privacy, anonymity, confidentiality, and accessibility. Further, HIVST can also help eradicate most of the barriers that stand in the way of people getting tested. It has the potential to be an acceptable option for high risk populations who would otherwise not test for HIV using currently available HCT services for various reasons, including stigma [6, 7]. All of these benefits of HIVST are aligned to recent World Health Organization (WHO) HIV testing guidelines that highlighted the potential contribution of HIVST to close critical gaps in HIV testing coverage worldwide [8].

The adoption and implementation of HIVST as a testing option in many African countries as well as globally did not come without challenges, criticism, and controversies from health experts and government stakeholders [2]. The main arguments against HIVST include the risks of inaccurate results, potential psychological risks posed by lack of adequate counselling, and uncertainty over unsupervised linkage to care for individuals who test positive for HIV [9, 10]. There is evidence that the arguments against self-testing are largely based on vague fears since HIVST is a new modality [11]. A recent review of articles showed little evidence of potential harm from selected self-tests as well as HIV self-tests [12]. However, the absence of face-to-face counselling, which is viewed as an essential component of all HCT models in South Africa, remains the most significant argument against HIVST [2]. It is therefore crucial that research on the acceptability and feasibility of HIVST should also investigate the provision of pre- and post-counselling.

South Africa has lagged behind in the adoption and creation of awareness of HIVST as a testing option to increase access to HIV testing mainly because of the constrains and arguments against HIVST. It is only recently that key stakeholders, engaged in HIV prevention and treatment, research, and policy formulation in South Africa, have indicated significant support for the introduction of HIVST in the country’s HCT portfolios [2]. Although HIVST is now acceptable in South Africa as an option to increase HIV testing [13], its acceptability among population groups with poor uptake of HCT remains limited. Therefore, future research on HIV testing acceptability is of significance to facilitate improvement of the uptake of HCT [14]. To investigate the acceptability of HIVST among students in Technical Vocational Education and Training (TVET)colleges, the study determined their HIV testing practices using the traditional provider and client initiated services, assessed their acceptability and willingness to use HIVST, and determined their perceptions on the provision of pre- and post-test counselling in the context of HIVST. South African youth are considered as one of the HIV high risk groups and TVET colleges enrol a large number of youth (at most 75%) of the youth from 16 to 24 years of age from poverty stricken backgrounds. TVET colleges are also a relatively new types of institutions (started after 2010) compared to generic institutions of higher learning such as Universities and hence they are under-researched while they enrol a cohort of students who are educationally and economically deprived like the majority of the people in South African communities [][15].

The study was conceptualised and conducted at a time when South Africa has not yet taken a position to promote and adopt HIVST. The findings of the study remain relevant even after the adoption of HIVST in the country, given the limited data on HIVST. The findings will highlight the gaps that still exist for scaling up HIVST and will also be used as a tool to increase social awareness of HIVST to further promote its adoption and uptake.

## Materials and Methods

### Study design

A formative evaluation design using the mixed approach was employed to assess the opinions of and acceptability of HIVST among TVET college students. Overall 16 colleges were invited to participate in the study, 13 colleges in Tshwane districts and three in Bojanala district. Since TVET colleges offer different types of qualification, to allow for variability of student participation by qualification type, all colleges were approached to participate in the study in order to minimize selection bias and to accommodate for refusal of institutional permission from college management. A survey was conducted in 12 TVET colleges after permission was granted by the college principals. Within each college, cluster sampling consisting of levels of study of the National Certificate Course (N1 to N6) was used to produce a representative sample of students. The research team obtained permission to randomly approach subject facilitators and administer the questionnaire. The population of students in each TVET college was about 10 000 students and a sample size calculated at 95% confidence level and 5% confidence interval was estimated at 370 students per college. The total number of students who volunteered to participate in the study were 3662.

This paper presents the quantitative findings regarding 12 TVET colleges in the Bojanala district in the North West Province and the Tshwane district in the Gauteng Province. Three colleges were from a rural district in the North West Province and the other nine were from an urban district in Gauteng Province. (TVET) colleges and offer vocational, occupational, and artisan education and training. The training offered in these colleges is classified as ‘post-school’ education, and the institutions train students who exited school after they complete the ninth grade. In South Africa, the TVET colleges were developed in 2012 as an addition to the generic tertiary or higher education institution. The colleges’ mandate is to focus on promoting skills development and vocational training. The purpose of these college system was designed to foster the new opportunity to the training of artisans and to build partnerships between educational institutions and employers and or the economic sector. The partnership between the TVET colleges and the employers enabled by involving Sector Education and Training Authorities (SETA) who are the intermediaries between the TVET colleges and the economic sector for purposes of ensuring learnership (skills-based training) placements in various sectors of employment. The role of the TVET initiative is to strengthen the contribution that public and private colleges play in further developing South Africa and allowing the South African labour force to better participate in the economy since vocational training aimed at helping the students to acquire the skills they need to go out into the job market and position themselves as skilled—rather than dispensable–labour. TVET colleges are also intended to address increasing levels of unemployment, particularly youth unemployment, and growing poverty [15, 16]. According to the audit-client report compiled by the Human Science Research Council of South Africa the national enrolment demographics of TVET colleges in in the country is 96% African black students, age distribution of the students shows that there are students of a younger age since up to 30% of students are older than 24 years of age, 0.2% disabled students [[15]]. The student population in TVET colleges come from families with poor backgrounds [[16]]

### Data collection

Data collection commenced from February to May 2015. The research team consisted of a research coordinator and five trained fieldworkers. Once permission was granted, a meeting was arranged with the campus principals of the TVET colleges after which the research team visited the colleges in order to explain the purpose of the study and discuss the protocol to be followed during the administration of the questionnaire. In all the colleges surveyed, classes were randomly assigned by the course facilitators to the research team, and all learners in the assigned classes were invited to participate. The purpose of study was explained to the students, and they were informed that participation was voluntary and that their responses were confidential. Informed consent was distributed to those who volunteered to participate. Anonymity was ensured by not gathering any personal information from the students. Students were also informed that they could withdraw from the study without any consequences at any time, should they wish to do so.

The study was conducted after obtaining ethical approval from the Research and Ethics Committee of the University of Limpopo, Medunsa Campus (MREC/H/46/2014: IR). Permission was obtained from the Department of Higher Education and Training, and the Head of Departments of District Higher Education and Training as well as the principals of the TVET colleges.

### Measures

The study instrument was a self-administered, semi-structured questionnaire that included the socio-demographic backgrounds of the learners, their sexual behaviours, HCT practices, and the acceptability of HIVST. We used a validated questionnaire from the Australian Secondary Students and Sexual Health Survey [17]. The questionnaire was adapted and validated and was used by the investigators to assess the acceptability of HCT in school [18]. For this survey, we added four main questions to assess the acceptability of HIVST as an option for testing. Students were asked about whether they would use HIVST, about their willingness to buy self-testing kits, about their opinions on the provision of pre- and post-testing for HIVST, and about information on how to use self-testing kits.

Students were asked if they used HCT in the past year, whether they were satisfied with HCT services, whether HIV testing was important for young people, and whether pre-test counselling for HIV was important.

To assess the acceptability and opinions of the students about HIVST, students were asked if they were aware of HIVST, whether they would use HIVST as a testing option, whether pre- and post-test counselling was necessary for HIVST, and whether they were willing to buy a self-test kit, to submit the test results for statistics. HIVST is part of the government strategy to increase uptake of HIV counselling and testing campaign, all test results whether positive or negative are to be submitted to be collated with the HCT statistics. They were also asked if they would confirm positive test results at a local clinic, if information leaflet would be adequate to explain how to use the self-testing kits and if the use of the toll-free hotline would be appropriate for pre- and post-test counselling.

Since the language of instruction in secondary schools, colleges and universities in South Africa is English, the questionnaire was developed and administered in English and took about 20 minutes to complete under the supervision of the research team to ensure completeness of data, but students were informed that they have the right not to answer any question that they were not comfortable answering.

### Data analysis

Descriptive statistics were presented as frequencies and proportion distribution for categorical variables. Bivariate analysis was performed to establish whether there was an association between gender and the variables describing sexual behaviour and the uptake of HCT. The unadjusted odds ratios (OR) found during bivariate analysis at a confidence level of 95% and the p-value of <0.05 were used to compute logistic regression using backward elimination. The dependent variables were ever tested for HIV and acceptability of HIVST. For logistic regression, we only included the statistically significant variables on unadjusted odds ratios from the bivariate analysis in order to limit the number of variables and to avoid unstable estimates. Data were analysed using STATA IC version 13.

## Results

The description of study participants in Table 1 illustrates the demographics, sexual behaviours and the HCT practices of the students. A total of 3,662 students participated, of which 68.28% were in years 1 to 3, while 31.72% were in years 4 to 6 of training. There were slightly more female students 56.6% than males. The mean age of the students was 21.9 years (SD: 2.7, age range 18–32 years). The majority of the students (*n* = 2,541, 70.4%) were between 20 and 24 years old.

**Table 1:** Demographics, sexual behaviour and testing practices of TVET students.

Variables	Frequency (N)	Percentage
**Gender (missing values = 57)**		
Female	2,040	56.6%
Male	1,565	43.4%
**Age group (missing values = 54)**		
Younger ≤ 24 years	3,127	86.7%
Older >24years	481	13.3%
**Age category (missing values = 54)**		
< 20	586	16.3%
20–24	2,541	70.4%
25–29	408	11.3%
≥ 30	73	2.0%
**Have a current sexual partner (missing values = 121)**		
Yes	2,894	81.7%
No	647	18.3%
**Sexually active (missing values = 60)**		
Yes	2,916	80.9%
No	686	19.1%
**Number of sexual partners in the past year (no missing values)**		
One partner	2,106	66.2%
Two partners	618	19.4%
More than two partners	458	14.4%
**Condom use during the last sexual act (no missing values)**		
Yes	1,801	66.5%
No	906	33.5%
**Ever tested for HIV (missing values = 24)**		
Yes	2, 698	72.2%
No	940	27.8%
**Know HIV status of sexual partner (no missing values)**		
Yes	2,130	60.2%
No	1,130	32.0%
Not sure	275	7.8%

More than three-quarters, 81.7% were had sexual relationships, 66.2% had one sexual partner, 19.4% had two sexual partners and 14.4% had more than two sexual partners. The mean age of sexual debut was 17.7 (SD: 2.4 years). Up to66.5% reported having used condoms in the last sexual act. Data also show that 72.2% of the participants had ever tested for HIV and 60.2% reported that they knew the HIV status of their sexual partners.

### Opinions of HIV counselling and testing and the reasons for uptake and non-uptake

Of the students who up took HCT, the services were accessed at the Clinic (48.6%), HCT Campaign (27.9%), at a hospital (11.7%), at a General practitioner (9.8%) and other unspecified service providers 2.0%). Data also show that 79.2% reported satisfaction with HCT services, and a majority of 91.1% considered HIV testing to be important for young people. In addition, 87.9% cited that HIV pre-test counselling was necessary (Table 2).

**Table 2:** Opinions on HCT and reasons for testing and not testing.

Variables	Frequency	Percentage
**Where HCT was done (n = 2,675)**		
Hospital	312	11.7%
inic	1,300	48.6%
General practitioner	262	9.8%
HCT campaign	747	27.9%
Other	54	2.0%
**Were you satisfied with VCT (n = 2,749)**		
Yes	2,177	79.2%
No	343	12.5%
Not sure	229	8.3%
**Pre-test counselling for HIV necessary (n = 3,591)**		
Necessary	3,158	87.9%
Not necessary	210	5.9%
Not sure	222	6.2%
**HIV testing important for young people (n = 3,457)**		
Important	3,148	91.1%
Not important	103	3.0%
Not sure	205	5.9%
**Reasons for HCT uptake (n = 2752)**		
To know my HIV status	2,097	76.2%
I was pregnant	196	7.1%
I am sexually active	128	4.7%
I was testing with my partner	178	6.5%
I was offered a test by a healthcare worker	152	5.5
**Reasons for HCT non-uptake (n = 1.048)**		
Not at risk of contracting HIV	265	23.5%
Not sexually active	220	19.5%
fear of stigma if the test results come out positive	168	14.9%
Not feeling comfortable testing in hospital settings	133	11.8%
Not wanting to know once HIV status	152	13.5%
Not sure if the results will be kept confidential	110	9.78%

From a sample of n = 2,752 who responded to reasons for the uptake of HCT, the student cited the reasons as wanting to know their status (76.2%), testing due to pregnancy (7.1%), being sexually active 128 (4.7%); tested with partner 178 (6.5%); and offered HCT by a healthcare worker 152 (5.5%). The reasons for the non-uptake of HCT from a sample of (n = 1048) were cited as not feeling at risk of contracting HIV 265 (23.5%); not being sexually active 220 (19.5) fear of stigma if the test results come out positive 168 (14.9%); not wanting to know once HIV status 152 (13.5%); not feeling comfortable testing in hospital settings 133 (11.8%); and not sure if the results will be kept confidential 110 (9.78%).

### Opinions and acceptability regarding HIVST

A proportion slightly less than half of the students who participated in the study (46.2%) we aware of HIVST prior to the survey. The acceptability of HIVST as a testing option was 87.1%. The proportion of students who indicated the necessity for pre-test counselling for HIVST was 60.7% and the necessity for post-test counselling for HIVST was 60.7%. Moreover, the willingness of participants to purchase a self-test kit was 74.7%, while the willingness to submit HIVST results at a local clinic for inclusion in the HCT statistics which is necessary for the targets of the HCT uptake campaign was at 14.8%, and the willingness to confirm HIV positive self-test results at a local health facility was 75.6%. In terms of the use of the toll-free hotline for provision of HIVST post-test counselling, only 40.0% indicated that hotlines were appropriate, while 45.7 were not sure and 14, 3% did not approve. Appropriateness of using information leaflets to provide information on how to use a self-test kit was accepted by47.9% of the students, 12.5% disapproved and 39.6% were not sure (Table 3).

**Table 3:** Opinions and acceptability of HIVST and counselling by gender.

Variables	Missing values	Male	Female	Total	*P* value
**Ever heard of HIVST before**					
Yes	83	722 (46.6%)	933 (46.1%)	1,659	0.818
No		832 (53.4%)	1,092 (53.9%)	1,933	
**Willing to use HIVST if it was available**					
Yes	88	1,203 (77.5%)	1,737 (85.9%)	2,947	0.000*
No		197 (12.7%)	173 (8.6%)	374	
Not sure		153 (9.8%)	111 (5.5%)	267	
**Pre-test counselling is necessary for HIVST**					
Yes	82	949 (61.0%)	1,229 (60.7%)	2,183	0.138
No		329 (21.1%)	473 (23.4%)	806	
No sure		278 (17.9%)	322 (15.9%)	605	
**Post-test counselling is necessary for HIVST**					
Yes	92	841 (54.2%)	1,162 (57.6%)	2,007	0.078
No		331 (21.3%)	420 (20.8%)	758	
Not sure		380 (24.5%)	436 (21.6%)	819	
**Willing to buy the self-testing kit**					
Yes	105	1,077 (69.8%)	1,584 (78.7%)	2,666	0.000*
No		258 (16.7%)	242 (12.0%)	504	
Not sure		208 (13.5%)	188 (9.3%)	399	
**Test with partner using HIVST kits**					
Yes	131	983 (63.9%)	1,594 (80.0%)	2,986	0.000*
No		320 (20.8%)	269 (13.5%)	301	
Not sure		235 (15.3%)	130 (6.5%)	268	
**Willing to submit HIVST results for health statistics**					
Yes	116	888 (73.3%)	1,211 (77.4%)	2,104	0.030*
No		220 (57.6%)	228 (60.2%)	451	
Not sure		432 (28.1%)	573 (28.4%)	1.011	
**Willing to confirm HIVST results in health facility**					
Yes	108	1,132 (73.3%)	1,155 (77.4%)	2,694	0.018
No		102 (6.6%)	119 (5.9%)	224	
Not sure		310 (20.1%)	336 (16.7%)	650	
**Telephone toll-free hotlines sufficient for post counselling**					
Yes	329	561 (38.6%)	774 (412%)	1,336	0.249
No		256 (13.6%)	256 (13.6%)	478	
Not sure		673 (46.3%)	850 (45.2%)	1,527	
**HIVST procedure provided on information leaflet is sufficient**					
Yes	139	707 (46.1%)	984 (49.5%)	1,693	0.098
No		188 (12.3%)	247 (12.4%)	441	
Not sure		638 (41.6%)	759 (38.1%)	1,403	

There were significance gender differences for acceptability of HIVST among the students, with more females showing acceptability versus males (*p* = 0.000), more female students willing to confirm HIV-positive test results at a health facility, more female students citing willingness to submit HIVST test results at the nearest health facility (*p* = 0.03), more females willing to use HIVST with their sexual partners (p = 0.000), and willing to buy HIVST kits (p = 0.000).

### Factors associated with the uptake of HIVST

The outcome measure for this study was the acceptability of HIV self-testing. Factors significant at the *p* < 0.05 level in unadjusted bivariate analysis were the following: being tested for HIV in the past year, being sexually active, number of sexual partners, ever tested for HIV, willing to buy HIVST, submit test results at the local health facility for HCT statistics, confirm test results at the local health facility and uptake HIVST with a sexual partner.

We first analysed computed the bivariate analysis followed by logistic regression to confirm the association. The outcome variable for this analysis was acceptability of HIVST. Logistics regression using forward elimination of the statistically significant variable for bivariate analysis was computed for the acceptability of HIVST.

Logistic regression (Table 4) showed that college students who were willing to uptake HIVST were at most four time more likely test using HIVST with their sexual partners at unadjusted odd ratio (OR = 3.99; CI: 2.84–5.62). At adjusted odd ration statistics show that college students who had ever tested for HIV within the HCT program were twice as likely to accept HIVST (OR = 2.13; CI:1.72–2.63).

**Table 4:** Factors associated with the acceptability of HIV self-testing.

Variables	Yes	No	N	p-value	Unadjusted OR (95% CI)	Adjusted OR (95% CI)
**Sexually active**			**3,573**			
Yes	2,418 (82.4%)	480 (75%)		0.000	1.30 (0.94–1.810)	1.20 (0.92–1.56)
No	515 (17.6%)	160 (25.0%)			Ref	
**Number partners**			**3,157**		** **	
One	1,768 (67.3%)	323 (61.2%)		0.000	Ref	
Two	512 (19.5%)	98 (18.6%)			1.15 (0.82–1.62)	0.87 (0.77–0.99)
More than two	349 (13.3%)	107 (20.3%)			0.74 (0.53–1.03)	
**Ever tested for HIV**			**3,574**			
Yes	2,286 (77.9%)	383 (59.9%)		0.000	1.38 (1.04–1.83)	2.13 (1.72–2.63)
No	659 (22.1%)	256 (40.1%)			Ref	
**Submit results for HCT statistics**			**3,546**			
Yes	1,826 (62.6%)	267 (42.5%)		0.000	1.47 (1.00–2.15)	0.96 (0.81–1.13)
No	328 (11.2%)	120 (19.1%)			Ref	
Not sure	763 (26.2%)	242 (38.4%)			1.15 (0.77–1.70)	
**Confirm results at a health facility**			**3,547**			
Yes	373 (58.9%)	2,307 (79.2%)		0.000	1.69.(0.59–1.64)	0.69 (0.57–0.86))
No	65 (10.3%)	157 (5.4%)			Ref	
Not sure	195 (30.8%)	450 (15.4%)				
**HIVST with partner**			**3,531**		** **	
Yes	2,229 (76.6%)	350 (56.4%)	3,531	0.000	3.99 (2.84–5.62)	0.58 (0.49–0.71)
No	470 (16.2%)	115 (18.5%)			Ref	
Not sure	211 (725%)	156 (25.1%)			1.66 (1.00–2.75)	

OR: Unadjusted odd ratio: CI -95% confidence interval; *significant p-value

## Discussion

The study assessed the acceptability of HIV self-testing among students in TVET colleges in rural and urban sub-districts of two provinces in South Africa in the wake of the adoption by many countries of HIV self-testing as an alternative HIV testing strategy. Since HIV self-testing is an alternative HIV testing option, we also assessed the students’ HIV testing practices using the available HCT services. The uptake of HCT was high among the students, with almost three-quarters (72%) indicating that they had been tested in the past year. The uptake of HCT is relatively high compared to that of students in colleges and universities in other countries [19–21]. Over three-quarters (76%) indicated that they tested to know their HIV status. Of the students (*n* = 1,048) who had not tested, about a quarter did not perceive themselves at risk of contracting HIV. This should be viewed in the context of reported risky sexual behaviours among the students. Over a third (33.3%) reported multiple sexual partners, and of the sexually active students, 33.5% did not use condoms the last time they had sex. Other reasons cited for not having been tested included not being sexually active, fear of stigma, not wanting to know one’s HIV status, not feeling comfortable testing in hospital settings, and not being sure that the results would be kept confidential. These findings are consistent with reasons cited in other studies [22, 23].

Even though less than half of the students were aware of HIVST prior to the survey, the majority (87.1%) considered HIVST to be acceptable, and 84% would uptake HIV self-testing with partners. Being sexually active, having multiple sexual partners, and having been tested for HIV were associated with acceptability of HIVST. A randomised control trial (RCT) by Choko and colleagues [24]. also showed high (84%) uptake of HIVST among people over the age of 16 years. This compares to the level of acceptability in our study. High HIVST acceptability has also been illustrated with different population groups in other studies in Sub-Saharan countries [25–27].

The cost of an HIV self-test kit has been identified as a potential barrier to adoption, willingness to use, purchase, and the uptake of HIVST, particularly among people in poor-resource settings [13, 28]. Despite the students being from poor-resource settings, we found that three-quarters (75%) reported the willingness to purchase self-testing kits. Willingness to self-test was also reported in a feasibility study conducted with university students in Canada; the acceptability of HIVST was also high among these students [29]. Nonetheless, about a quarter of the students in the current study were not willing to purchase the self-testing kit. This suggests that if there was no cost involved in the acquisition of self-testing kits, the uptake of HIVST might greatly increase [13]. While most trainee nurses in a South African study were willing to purchase the self-testing kits, some felt that it should be provided for free because the cost will be a potential barrier for the uptake of HIVST [30].

We found that 59%% of the students indicated that they would submit their HIVST test results at a local clinic for the purpose of health statistics even though less than half of the sample participating in the study did not know about HIVST prior to the survey. The HIVST is regarded as safe if implemented correctly to ensure the accuracy of the tests, and there is confirmation testing and referral for counselling and care [9, 29, 30]. According to Estem and colleagues [13], setting up effective strategies for measuring HIV incidence and prevalence and the extent to which people who have conducted the HIVST report their status is one of the conditions that should be addressed before the scale up of HIVST.

In terms of confirming positive test results, over three-quarters (75%) reported the intention to confirm their HIV positive self-test results at a local health facility. While it is commendable that the majority wanted to be linked to a health facility after self-testing, currently, there are no referral mechanisms to link people who self-test to treatment and care [2]. Some of the suggestions on the linkage to confirmation testing included that persons with positive HIVST results return to their pharmacist where the self-testing kits were purchased to seek advice [30, 31]. Makusha and colleagues [2] suggested that the self-testing instruction kits should provide adequate information on what do after self-testing. Despite high intentions to confirm HIVST results, a quarter (25%) of the students did not intend to confirm their self-test results. Willingness to confirm HIV results and seek treatment after a self-test is key to effective control of the HIV epidemic [2]. The role of educating people on what to do after self-testing cannot be over emphasised, particularly because HIVST requires individuals to be more proactive than when testing using facility-based HCT approaches [31].

In order to ensure proper implementation of HIVST, post-test counselling should be accessible to people who conduct HIVST [2, 6, 30, 32], and the reported satisfaction level of 79.4% with the current HCT among the students explains the importance of face-to-face counselling. Makusha and colleagues [2] argued that the absence of face-to-face counselling remains a significant challenge for the adoption and implementation of HIVST. This is true of the findings in the current study; we found that the suggestion that pre-and post-counselling be provided through an anonymous toll-free hotline service was acceptable to only 40% of the students. Pai and colleagues [33] found diversity in opinions as to the desired approach to counselling. Only 16% of the students preferred anonymity, and 41% were comfortable with either anonymity or face-to-face counselling, while 39% preferred face-to-face counselling. In our study, similar preferences for face-to-face counselling were reported as in other studies [26, 32].

In spite of recommendations and suggestions that self-testing instruction kits should provide information about self-testing in the form of leaflets [2, 4, 27–31]], only half (50%) of the students in the current study felt that information contained in the leaflet would be sufficient to provide information on how to use self-testing. This implies that other strategies and approaches should be explored to educate and promote self-testing. Besides pamphlets or leaflets, a link to a YouTube video demonstration was suggested as one of the ways to show people how to use self-testing kits [30]. The use of instructional videos and pamphlets in a feasibility study resulted in successful conducting of self-tests among students in an unsupervised HIVST programme [29].

## Limitations

Several shortfalls of this study should be considered when interpreting the findings. The study employed a cross-sectional survey design with only college students in two provinces included in the sample; hence, there is a level of selection bias even though probability sampling was used to select the sites (TVET colleges), and random sampling was done to address the bias. Since probability sampling was used and the sample size was considerable, the findings can be generalised to other similar contexts but not to the general population of young people in South Africa. There were incomplete responses in some of the questions, which resulted in missing values, but the sample size in the analysis was adjusted to account for the missing values.

## Implications

Currently, HIV self-testing is acceptable in South Africa as an HIV testing option, and self-tests kit are available over the counter in pharmacies. Nonetheless, the high acceptability of HIVST among the students calls for extensive planning and preparation for the scaling up of HIVST beyond its availability in pharmacies. Even though the students intend to confirm their HIV positive results with a local clinic, there is currently no plan or system for linking HIVST to health facilities for confirmatory testing and HIV care as recommended by WHO. In planning for linkages, the country should guard against supervised HIVST because the fundamental principal for HIVST is to allow choice for users to test without the need for a health worker to be present [32]. For a country with such diverse populations, the provision of HIVST should take into consideration the context of the target populations and tailor the distribution site, cost, and support accordingly. This will address the needs of population groups who may specifically choose HIVST to avoid the need for counselling that does not meet their needs, who may afford to pay for self-testing kits, and who might know when and how to access treatment and care. A one-size-fits-all approach may not be appropriate for South Africa. The attempt to follow up on the usage of HIVST at government clinics should not represent a barrier that could undermine the principal motivation for those seeking self-testing, namely full confidentiality [32].

There is no doubt that pre- and post-test counselling remains a challenge for the scaling up of HIVST. The remote services that will complement the convenience of HIVST, such as toll-free hotlines, are vital for the scaling up and implementation of HIVST. However, the telephone hotline as a counselling approach has not been tested in South Africa in an unsupervised HIVST model, and our findings suggest that this is not the preferred method of counselling for the students. The appropriateness of this counselling approach should be tested in unsupervised feasibility studies to explore user perspectives on the counselling and linkage to care requirements in the self-testing model [6].

The current results should be taken in the context that these are college students and the telephone hotlines might work for other population groups. Nevertheless, there is a need for further studies to explore provision of counselling for effective implementation of HIVST in South Africa and other African countries. Awareness campaigns similar to those conducted to promote provider-initiated testing and counselling should be considered to educate communities about HIVST.

## Conclusions

A majority of the TVET college students already utilise HCT in public health facilities, and a vast majority have high levels of acceptability for HIVST and intention to test with partners. The results suggest that scaling up HIVST in South Africa is feasible, considering the majority of students are willing to purchase HIVST kits despite being from low socioeconomic settings and to confirm HIV positive test results at a local health facility. It is also evident that HIVST as a new modality is more acceptable to the students who had ever tested for HIV in the existing HCT program. This means that there is a need for programme designers to device innovative ways of making new modalities such as HIVST more appealing to those who never up took HCT for various reasons.

## Supporting Information

S1 Appendix Spreadsheet of minidataset for TVET colleges. (XLSX)
